# Impact of COVID-19 social distancing recommendations on pulmonary function, nutritional status, and morbidity in patients with cystic fibrosis

**DOI:** 10.1590/1984-0462/2024/42/2022198

**Published:** 2023-08-25

**Authors:** Maria Amélia Bagatini, João Paulo Heinzmann-Filho, Fernanda Maria Vendrusculo, Leonardo Araújo Pinto, Márcio Vinícius Fagundes Donadio

**Affiliations:** a Pontifícia Universidade Católica do Rio Grande do Sul, Porto Alegre, RS, Brazil.; b Centro Universitário Cenecista de Osório, Osório, RS, Brazil.; c Universitat Internacional de Catalunya, Barcelona, España.

**Keywords:** Cystic fibrosis, Social isolation, Pulmonary function, Nutritional status, Morbidity, SARS-CoV-2, Fibrose cística, Isolamento social, Função pulmonar, Estado nutricional, Morbidade, SARS-CoV-2

## Abstract

**Objective:**

To evaluate the impact of COVID-19 social distancing recommendations on nutritional status, pulmonary function, and morbidity in patients with cystic fibrosis (CF).

**Methods:**

A retrospective cohort study including patients older than six years with a diagnosis of CF was performed. Demographic and clinical data, anthropometric measurements, pulmonary function, days of antibiotic use, and length of hospital stay were recorded. Variables were recorded at three time points relative to the baseline for implementation of social distancing measures: T-1 (14 months before implementation), T0 (baseline), and T1 (14 months after implementation). Delta (Δ) was calculated for each period: Δ_1_ (pre-pandemic T0-T-1) and Δ_2_ (pandemic T1-T0).

**Results:**

The study included 25 patients, with a mean age of 11.7±4.3 years. The mean forced expiratory volume in the first second (FEV_1_) was 85.6±23.6%, and body mass index (BMI) was 17.5±3.0 kg/m^2^. When comparing the two periods (Δ_1_ and Δ_2_), there was a significant increase in the FEV_1_/forced vital capacity (FVC) ratio (p=0.013) and in the forced expiratory flow between 25 and 75% of vital capacity (FEF_25–75%_) (p=0.037) in the pandemic period. There was also a significant reduction (p=0.005) in the use of antibiotics in the pandemic period compared with the pre-pandemic period. The Δ_1_ and Δ_2_ values did not differ significantly for BMI, FEV_1_, or length of hospital stay.

**Conclusions:**

COVID-19 social distancing recommendations had a positive impact (decrease) on morbidity (use of antibiotics) and small airway obstruction (FEF_25–75%_) in patients with CF.

## RESUMO

**Objetivo:**

Avaliar o impacto das recomendações de medidas de distanciamento social por COVID-19 sobre estado nutricional, função pulmonar e morbidade em pacientes com fibrose cística (FC).

**Métodos:**

Estudo de coorte, retrospectivo, que incluiu pacientes com diagnóstico de FC e idade superior a seis anos. Foram registrados os dados demográficos, antropométricos, clínicos, de função pulmonar e o total de dias de uso de antibiótico e de hospitalizações. As variáveis foram registradas em três momentos relativos ao início das recomendações de distanciamento social: T-1 (14 meses antes), T0 (início das recomendações) e T1 (14 meses depois). Foram calculados deltas (Δ) para cada um dos períodos: Δ_1_ (pré-pandemia T0-T-1) e Δ_2_ (pandemia T1-T0).

**Resultados:**

Vinte e cinco pacientes, com média de idade de 11,7±4,3 anos, sendo 76% homozigotos para Δf508 e 28% colonizados por *Pseudomonas aeruginosa*, foram incluídos. A média do volume expiratório forçado no primeiro segundo (VEF_1_) foi de 85,6±23,6 (% do previsto) e o índice de massa corporal (IMC) foi de 17,5±3,0 kg/m^2^. Ao compararmos os períodos (Δ_1_ e Δ_2_), houve aumento significativo do VEF_1_/CVF (p=0,013) e do FEF_25–75%_ (p=0,037) no período das recomendações de distanciamento. Também se observou redução significativa (p=0,005) do uso de antibióticos no período da pandemia em comparação ao período anterior a ela. Não houve diferenças significativas nos deltas para o IMC, VEF_1_ e dias de hospitalização.

**Conclusões:**

As recomendações de distanciamento social por COVID-19 tiveram impacto positivo (redução) sobre a morbidade (uso de antibióticos) e a obstrução de vias aéreas de menor calibre (FEF_25–75%_) em pacientes com FC.

## INTRODUCTION

In December 2019, China informed the World Health Organization (WHO) of the outbreak of a novel coronavirus (SARS-CoV-2).^1^ Brazil reported the first cases of SARS-CoV-2 infection in February 2020, and the number of infected individuals increased steadily^1^. In January 2020, given the large number of people infected with SARS-CoV-2 inside and outside China, the WHO declared the outbreak of a public health emergency of international concern, later referred to as the COVID-19 pandemic.^2^

International health authorities have implemented several measures to control the pandemic, with some regional differences in their implementation. The measures included 1.5 to 2 meters of social distancing, closure of schools, universities, nonessential businesses, and public leisure spaces, and quarantine requirements^3^ in order to prevent and control the spread of the virus, particularly in high-risk groups, including patients with chronic respiratory diseases.^1,2^

Patients with cystic fibrosis (CF) required special care, as they would be more vulnerable to COVID-19 due to preexisting abnormalities in body systems, including the respiratory tract.^4^ The accumulation of secretions in the lungs increases susceptibility to respiratory tract infections, leading to loss of pulmonary function, reduced exercise tolerance, and hospitalization.^5,6^ Acute pulmonary exacerbations are a major cause of hospitalization in patients with CF.^6^ Frequent respiratory tract infections associated with exacerbations can accelerate the progressive loss of pulmonary function, contributing negatively to increased morbidity and mortality.^7^ The number of hospital admissions and the use of antibiotics are important markers of morbidity.^8,9^ In addition, nutritional changes also play an important role in the disease. A 10% increase in body mass index (BMI) has been shown to be associated with a 4% increase in pulmonary function in underweight children with CF.^10^ Furthermore, low BMI has been associated with an increased likelihood of hospitalization/morbidity, prolonged hospital stay, and increased risk of death.^7,9,11^

Recent data show that 40% of children with CF have engaged in less physical activity during the pandemic, and 40.3% of the families of children with CF have changed their eating habits.^12^ Additionally, a study showed that COVID-19 confinement restricted free movement, which negatively affected maximal oxygen uptake in adolescents with CF.^13^ Brazilian authorities have issued state and local decrees to enforce measures aimed at preventing SARS-CoV-2 infection.^14^ The most important measures to reduce viral transmission and to control the pandemic outbreak include the publication of information bulletins, social distancing (1.5 to 2 meters), temporary closure of schools and nonessential businesses, recommendations to sanitize hands more frequently, use of alcohol-based sanitizers and face masks, restrictions on public transportation, and cancellation of public gatherings.^1,3^

However, the effects of these recommendations on pulmonary function, nutritional status, and clinical morbidity in patients with CF have not yet been investigated. Therefore, the current study aimed to evaluate the impact of COVID-19 social distancing recommendations on pulmonary function, nutritional status, and morbidity in patients with CF. We hypothesized that a higher level of social isolation and less exposure to infectious agents would contribute to improving clinical markers.

## METHOD

This retrospective cohort study analyzed a convenience sample of patients meeting the eligibility criteria described below. The study included all patients older than six years, of both sexes, with a genetic diagnosis of CF who were regularly followed up at a CF Referral Center. All participants were required to have demographic and clinical data, nutritional status, and pulmonary function records at three time points relative to the baseline (T0) for implementation of social distancing measures: before implementation (T-1 — January 2019), at baseline (T0 — March 2020), and after implementation (T1 — June 2021). Patients with inaccurate or incomplete medical records and those who had experienced an exacerbation in the 21 days prior to data collection were excluded. Medical records with death information were also excluded from the study. The Research Ethics Committee of the Pontifícia Universidade Católica do Rio Grande do Sul (PUCRS), Brazil, approved the study (No. 48404821.7.0000.5336).

A multidisciplinary team is responsible for the CF outpatient clinic. At each visit, patients undergo clinical evaluation, pulmonary function tests, and collection of oropharyngeal swabs or sputum samples for culture. Information collected from the medical records included demographic and clinical data (age, sex, ethnicity, genetic mutation, pancreatic insufficiency, and bacterial colonization of the airways), nutritional status (height, body mass, and BMI), pulmonary function (spirometry), and morbidity (number of days in hospital and number of days using oral and/or intravenous antibiotics). The research team reviewed all the data to ensure the quality of the records.

**Figure 1:**
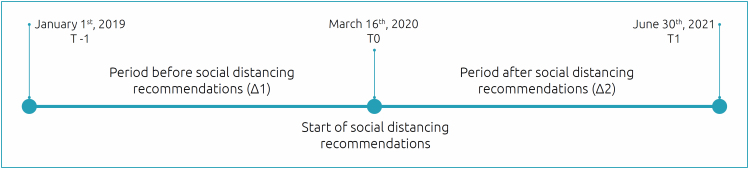
Timeline of periods used to analyze and compare lung function, nutritional status and morbidity variables in patients with cystic fibrosis.

All data were collected at three different time points (Figure 1). The first time point (T-1) was approximately 14 months before the implementation of social distancing measures, the second (T0) was as close as possible to the implementation of such measures, and the third (T1) was approximately 14 months after. The behavior of the data collected between T-1 and T0 was used as a control for comparison with the data collected between T0 and T1 (social distancing period).

Anthropometric assessment included measurements of weight and height in triplicate or until two equal values were obtained. With the patient standing upright, weight was measured on a digital scale (G-tech, Glass 1 FW, Rio de Janeiro, RJ, Brazil) and height was measured with a portable stadiometer (AlturaExata, TBW, São Paulo, SP, Brazil). BMI was then calculated (weight [kg]/height^2^ [m]) and expressed as both an absolute value and a z score, adjusted for age. Pulmonary function was assessed using a Koko spirometer (PDS Instrumentation, Inc., Louisville, CO, USA). The main spirometric parameters were forced expiratory volume in the first second (FEV_1_), forced vital capacity (FVC), FEV_1_/FVC ratio, and forced expiratory flow between 25 and 75% of vital capacity (FEF_25–75%_). All procedures were performed in accordance with the American Thoracic Society criteria.^15^

For statistical purposes, quantitative data were tested for distribution normality using the Shapiro-Wilk test and expressed as a mean (standard deviation — SD) or median (interquartile range — IQR) according to their distribution. Qualitative data were expressed as absolute and relative frequencies. Delta (Δ) was calculated for the period from T-1 to T0 (Δ_1_ — control) and for the period from T0 to T1 (Δ_2_ — social distancing). Quantitative data obtained before and during the social distancing period were compared using paired Student’s *t* test or Wilcoxon’s test according to their distribution. Data were analyzed in Statistical Package for the Social Sciences (SPSS), version 18.0, and the significance level was set 5% (p<0.05) for all analyses.

## RESULTS

**Figure 2:**
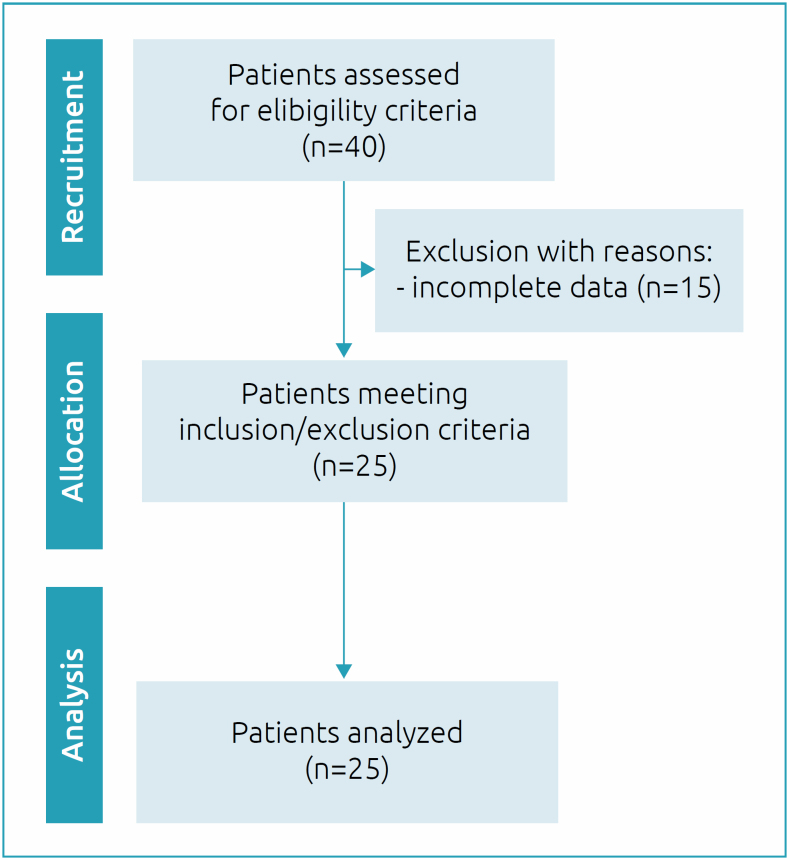
Flowchart of recruitment, selection and analysis of patients included in the study.

A total of 40 patients were screened, 15 of whom were excluded on the basis of our inclusion/exclusion criteria, including unavailability of data for the study period, age, missing data, and ongoing diagnosis. Figure 2 shows the flow diagram of participant selection. The study included 25 patients, with a mean age of 11.7±4.3 years; 64% were male. Regarding genetics, 76% were homozygous for the Δf508 mutation and 28% had chronic *Pseudomonas aeruginosa* colonization. The mean BMI was 17.5±3.0 kg/m^2^ and the z-score was 0.2±0.9, indicating a slightly nutritionally compromised sample. The mean FEV_1_ was 85.6±23.6% and the mean FVC was 89.8±20.0% (Table 1).

**Table 1:** Characterization of the studied sample.

Variables		n=25
Demographics		
	Age (years)	11.7±4.3
	Male, n (%)	16 (64)
Anthropometric		
	Weight (kg)	33.1±15.5
	Height (cm)	133.2±20.9
	BMI (kg/m^2^)	17.5±3.0
	BMI (z-score)	0.2±0.9
Genotypic		
	F508del homozygote, n (%)	19 (76)
	F508del heterozygote, n (%)	6 (24)
Pancreatic insufficiency		
	Yes, n (%)	25 (100)
Chronic colonization of the airways		
	Pseudomonas aeruginosa, n (%)	7 (28)
	Staphylococcus aureus, n (%)	19 (76)
	Haemophilus influenzae, n (%)	2 (8)
Lung function		
	FEV_1_ (L)	1.5±0.6
	FEV_1_ (% of predicted)	85.6±23.6
	FVC (L)	1.9±0.9
	FVC (% of predicted)	89.8±20.0
	FEV_1_/FVC (absolute)	0.9±0.0
	FEV_1_/FVC (% of predicted)	94.0±11.3
	FEF_25–75%_ (L.min^−1^)	1.8±0.9
	FEF_25–75%_ (% of predicted)	80.6±38.7

BMI: body mass index; FEV_1_: forced expiratory volume in the first second; FVC: forced vital capacity; FEF_25-75%_: forced expiratory flow between 25 and 75% of vital capacity; cm: centimeters; kg: kilogram; L: liters. Values expressed as absolute (relative) frequency or mean ± standard deviation, as indicated.

When comparing BMI and pulmonary function (FEV_1_ and FVC) between T−1, T0, and T1, there was no significant difference between mean values or Δ_1_ and Δ_2_ values for absolute BMI (Table 2). However, the FEV_1_/FVC ratio (*p*=0.013) and FEF_25–75%_ (p=0.037) increased significantly in the social distancing period (Table 2).

**Table 2:** Comparison of body mass index and lung function between pre- and post-social distancing recommendations.

Variables	T-1	T0	T1	Δ_1_	Δ_2_	p-value
BMI (absolute)	17.5±3.0	18.4±2.8	19.3±3.5	0.8±1.2	0.9±1.4	0.950
BMI (z-score)	0.2±0.9	0.3±0.8	0.3±1.0	-0.0±0.4	-0.1±0.6	0.708
FEV_1_ (%)	85.6±23.6	82.7±24.5	80.6±22.5	-2.8±12.1	-2.1±20.1	0.891
FVC (%)	89.7±20.0	88.9±23.6	85.2±21.8	-0.8±13.8	-3.7±18.8	0.611
FEV_1_/FVC (absolute)	0.8±0.1	0.8±0.9	0.8±0.1	-0.03±0.0	0.02±0.0	0.013*
FEF_25-75%_ (%)	80.5±38.7	71.6±37.1	74.6±29.8	-8.9±17.5	3.0±23.6	0.037*

T-1: assessment prior to distancing recommendations; T0: assessment at the beginning of distancing recommendations; T1: assessment after distancing recommendations; BMI: body mass index; FEV_1_: forced expiratory volume in one second; FVC: forced vital capacity; FEV_1_/FVC: ratio between FEV_1_/FVC; FEF_25–75%_: forced expiratory flow between 25–75% of vital capacity; Δ1: difference between T0 and T-1 assessments (pre-pandemic period); Δ_2_: difference between T1 and T0 assessments (pandemic period). Data expressed as mean and standard deviation. *Indicates significant value (p<0.05) in the comparison between Δ_1_ and Δ_2_.

**Figure 3:**
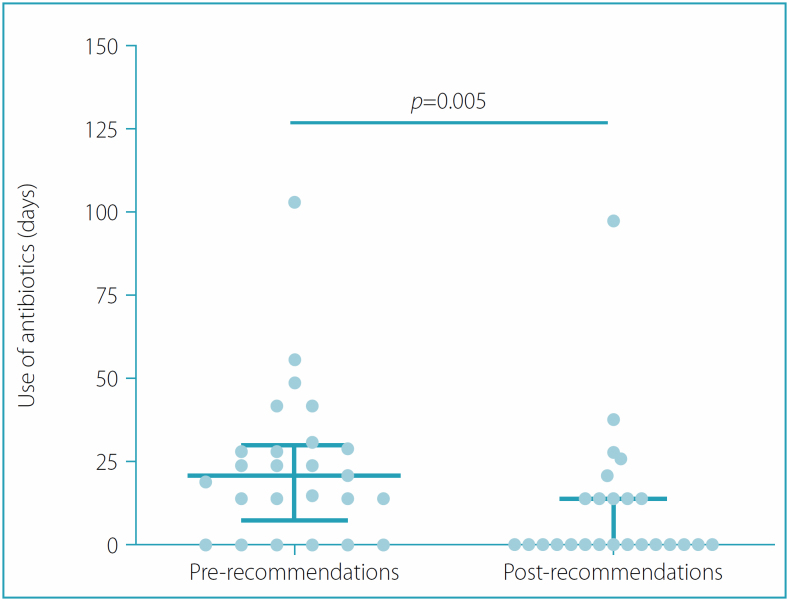
Comparison of antibiotic use between pre- and post-social distancing recommendations.

Regarding morbidity, Figure 3 shows that there was a significant reduction (p=0.005) in the use of antibiotics in the social distancing period (0.0 [0.0–14.0]) compared with the period before the implementation of social distancing measures (21.0 [7.0–30.0]). However, there was no significant difference (p=0.628) in total days of hospital stay.

## DISCUSSION

The findings of the present study showed an association between COVID-19 social distancing measures and improvement in pulmonary function, especially in small airway obstruction. In addition, these measures had a positive impact on morbidity with reduced use of antibiotics in patients with CF. However, nutritional status remained unchanged. These results can contribute to the development or maintenance of measures aimed at reducing morbidity and improving the quality of life of patients with CF.

Social distancing measures have brought sudden and abrupt changes to society as a whole, with the adoption of several preventive measures such as reduced urban mobility, closure of schools and nonessential businesses, use of face masks, recommendations for frequent hand washing, use of alcohol-based hand sanitizers before and after direct contact with objects and/or people, and distancing of 1.5 meters or more between people(1). Despite some regional variations in the implementation of measures, social distancing had an impact on vehicle mobility,^16^ reducing the rates of nitrogen dioxide (NO_2_) from 14.31 to 8.61 ppb and carbon dioxide (CO_2_) from 0.49 to 0.22 ppm.^17^

Altogether, this series of direct or indirect measures could have an impact on the respiratory health of patients with chronic diseases, such as CF. Evidence from patients with CF shows that pulmonary function, measured by spirometry, is directly associated with survival,^18^ thus representing an important and reliable marker of disease progression. Studies have shown that the decline in lung function occurs gradually and the annual rate of FEV_1_ decline varies between 1.0 to 2.5% in children and adolescents.^19–21^ This is in accordance with the annual rate of decline found in the present study (approximately 3%). In addition, present data showed a significant increase in the FEV_1_/FVC ratio and FEF_25–75%_ in the social distancing period, without a decrease in other parameters during this period. These measures are sufficiently sensitive to assess the onset of pulmonary function decline, which is characterized by the involvement of more peripheral airways.^21^ This improvement may be associated with more time spent on health-related self-care during social isolation, thereby increasing the time spent on treatment as a whole,^22^ including inhalation therapy, oral medications, adequate nutrition, and physical therapy. In addition, the increase in FEF_25–75%_ and no reduction in other parameters, such as FEV_1_, may also be related to closer supervision by family members and less exposure to infectious agents in general.^23,24^

Nutritional status as well as respiratory tract conditions are considered major cornerstones in the follow-up and treatment of patients with CF.^25,26^ Previous studies investigating COVID-19 and nutritional status showed that most participants with CF were able to maintain or had only a slight increase in weight during social distancing.^27^ Our observations are, at least in part, consistent with these findings as our patients had no significant increase in their nutritional status. Also, our patients showed no weight reduction, which was an important finding, since nutritional status is associated with risk of hospitalization and prognosis.^5,7^

The duration of antibiotic use has been associated with pulmonary function decline in patients with CF.^8,9^ Evidence has also shown that the number of hospital admissions is associated with disease morbidity.^6^ Therefore, the duration of antibiotic use and length of hospital stay are markers of disease morbidity/mortality. In the present study, social distancing measures had a positive impact on reduced antibiotic use during the pandemic, which may be related to less exposure to infectious agents as a result of the closure of schools and nonessential businesses. A recent study in a pediatric emergency department demonstrated a significant decrease of more than 70% in the airborne or fecal-oral transmission of infectious diseases, including bronchiolitis, common cold, gastroenteritis, and acute otitis.^28^ Another study reported a 70% decrease in pediatric visits and hospitalizations in 2020, although COVID-19 has considerably increased the mortality rate from acute respiratory distress syndrome in Brazil.^29^ However, in the present study, no changes were observed in the need for hospitalization during the study period. Although there is no single explanation for the maintenance of these numbers, even with social distancing measures, a potentially related factor is the admission of patients with more severe disease.

This study has limitations, including its retrospective design, as obtaining data from secondary databases might have increased the number of exclusions (37.5%) due to missing data, and the convenience sample recruitment, which may affect data generalizability. In addition, the reduced number of patients chronic colonized by *Pseudomonas aeruginosa* and the good nutritional status of the sample may have positively contributed to the results found. The lack of data on specific exacerbation signs may also be considered as a limitation of the present study.

In conclusion, our results showed that COVID-19 social distancing recommendations had a positive impact (decrease) on morbidity (use of antibiotics) and small airway obstruction (FEF_25%-75%_) in patients with CF. These data can contribute to a better understanding of how the pandemic and restrictive measures have affected the clinical outcomes of patients with CF, allowing the implementation of more effective prevention and treatment strategies.

## Data Availability

The database that originated the article is available with the corresponding author.
